# Effect of Hot Weather on Fatigue

**Published:** 1919-11-22

**Authors:** 


### EFFECT OF HOT WEATHER ON FATIGUE.

The equilibrium of the human machine is so delicately poised that its metabolism is at once affected by any abnormal condition to which it is subjected, though, at the same time it must be remembered that its intricate machinery can, in health, readily adapt itself to varying circumstances.

During a spell of intense heat, such as we experienced a short time ago in this country, the balance of work usually maintained between the three great excretory organs—viz., the lungs, kidneys, and skin, is upset, often to a marked degree; that is to say, the amount of sweat, which contains urea, etc., and which is excreted by the sweat-glands of the skin, is markedly increased; whilst the urine extracted from the blood by the kidneys, and which contains by far the greater proportion of the urea synthesised in the body, is correspondingly diminished.

### Scientific Ventilating.

As Dr. Leonard Hill in his article on "Scientific Ventilation" points out, the heart undergoes much extra fatigue during great heat, due to the double strain of carrying on its ordinary work, and at the same time cooling the body. The effect of such high temperatures on the output of mental and physical work is seriously to diminish it, and especially is this the case when the body of the worker is subjected to inefficient ventilation. It is a well-known fact to everyone that a feeling of lassitude pervades all the mental and physical systems, and work of any kind becomes a much more laborious proceeding. Physiologists have shown that there is a heat-regulating centre in the regions of both corpora striata in the brain, and also that sensations of temperature from the surfaces of the body are conveyed from these areas by the afferent, or sensory, nerves to the spinal cord, whence they are transmitted *via* the spino-thalamic fasciculi to the special nuclei in the brain. There is, therefore, a set of machinery devised by Nature to cope with climatic changes. We must not forget, also, that a special collection of nerve-fibres convey vaso-motor impulses to the coats of the blood-vessels, and to many other organs, etc., and thus themselves take a large share in the regulation of surface temperature. To state the matter briefly several practical points should be carefully considered which can help to lessen the ill-effects of very high temperatures upon the mental and physical physique.

### Some Practical Points.

First, it is essential that clothing should be light and porous; for instance, cellular underwear is much more beneficial in this respect than silk or cotton. Secondly, that food should be of a less stimulating nature than is the case in colder temperatures. Meat, which contains a large proportion of protein, and therefore nitrogenous compounds, ought to be taken sparingly. Again, alcohol, which by its chemical properties throws more work on the heart and excretory organs, is not by any means an ideal beverage under these conditions. It should, however, be noted that alcohol, by its action in causing dilatation of the peripheral arterioles, does aid in throwing off a goodly proportion of heat from the surface of the body; but it has been proved that the advantages derived from its use in hot weather are far outweighed by the disadvantages. Thirdly, a moderate amount of exercise is beneficial, but strenuous athletics should be discouraged. Fourthly, it is vitally necessary that there should be a very efficient system of ventilation in all dwellings and workshops. Carbon dioxide has a marked narcotic effect upon the central nervous system, and this effect will be greatly augmented by the addition of excessive heat; hence the value of fresh air cannot be too much emphasised.

